# Flexible X‐Ray Detector for Cumulative Dose Monitoring Through Reversible Photochromism and Luminescence Modulation

**DOI:** 10.1002/advs.202412986

**Published:** 2024-12-23

**Authors:** Xue Bai, Yueteng Zhang, Heping Zhao, Yingzhu Zi, Zan Xu, Anjun Huang, Yangke Cun, Yue Liu, Zhiguo Song, Jianbei Qiu, Jiayan Liao, Zhengwen Yang

**Affiliations:** ^1^ College of Materials Science and Engineering Kunming University of Science and Technology Kunming 650093 China; ^2^ Southwest United Graduate School Kunming 650092 China; ^3^ Institute for Biomedical Materials and Devices (IBMD) Faculty of Science University of Technology Sydney Sydney NSW 2007 Australia

**Keywords:** luminescence modulation, photochromism, X‐ray detection, X‐ray imaging

## Abstract

This study presents a multi‐mode X‐ray detection and imaging strategy by integrating photochromism, photoluminescence, and radioluminescence into Tb^3+^‐doped CaAl_2_Si_2_O_8_. CaAl_2_Si_2_O_8_: Tb^3+^ exhibits stable radioluminescence, oxygen vacancy‐related photochromism, and photoluminescence modulation, all of which showed a linear relationship with X‐ray exposure. This multi‐mode response enables high‐quality imaging and detection in both bright and dark conditions, facilitating time‐dependent cumulative X‐ray radiation dose assessments. Reversible color and luminescence changes are achieved through cyclic tests involving alternating X‐ray and 473 nm laser irradiation. The PDMS CaAl_2_Si_2_O_8_: Tb^3+^ ink and flexible film demonstrate high suitability for wearable X‐ray detection devices and imaging of irregular objects, offering an innovative approach to X‐ray detection and imaging.

## Graphical abstract

Multi‐mode X‐ray detection and imaging are achieved in CaAl_2_Si_2_O_8_: Tb^3+^ through photochromism, photoluminescence, and radioluminescence. The material is sensitive to cumulative X‐ray doses, with photochromism and luminescence modulation showing a linear dependence on X‐ray exposure conditions. This makes it suitable for wearable X‐ray detection and imaging of irregular objects.

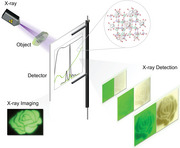

## 1 Introduction

The exceptional penetration capability of high‐energy particles has made X‐ray imaging technology a widely adopted method in security inspections, medical diagnostics, environmental testing, and radiation detection.^[^ ^1^ ^]^ As the demand for various applications increases, there is a growing need for the development and iterative design of X‐ray detectors that offer low detection limits, high stability, and high spatial resolution. Direct X‐ray detection using semiconductors like CdTe and CdZnTe provides high energy and spatial resolution but faces challenges such as non‐uniform charge transport, significant noise, and high operating voltage.^[^ ^2^ ^]^

Scintillators present an alternative by converting high‐energy X‐rays into low‐energy UV–VIS photons, which can then be detected by photodetectors.^[^ ^3^ ^]^ Organic scintillators offer optical advantages for manufacturing large‐area flexible electronic devices. However, the presence of light elements (C, H, O, N) results in low X‐ray absorption and limited exciton utilization efficiency, and the introduction of heavy atoms may cause emission quenching.^[^ ^4^ ^]^ Conventional inorganic scintillators, such as CsI: Tl, Lu_1.8_Y_0.2_SiO_5_: Ce, PbWO_4_, and Bi_4_Ge_3_O_12_, require complex crystal growth processes.^[^ ^5^ ^]^ Metal halide perovskites are susceptible to decomposition when exposed to high humidity, elevated temperatures, and high‐energy radiation, coupled with the risk of heavy metal toxicity.^[^ ^6^ ^]^ Additionally, traditional inorganic scintillators are widely used for planar imaging and are primarily ideal for real‐time monitoring. However, they are unsuitable for flexible imaging and the assessment of cumulative X‐ray radiation doses over an extended period.^[^ ^7^ ^]^ Persistent luminescent materials offer a promising solution for X‐ray energy storage and delayed information readout. For example, Tb^3+^‐activated NaLuF_4_ nanocrystals, Tb^3+^@NaMgF_3_, and NaLuF_4_: Gd/Tb show potential for enhanced persistent luminescence, but challenges in controlling the traps hinder the stability of X‐ray information release after radiation removal.^[^ ^8^ ^]^

Previous studies have shown that certain inorganic phosphors exhibit reversible changes in color and optical properties when exposed to alternating irradiation between X‐rays and visible light or heat treatment, offering significant advantages in X‐ray detection and imaging.^[^ ^9^ ^]^ The photochromic effect and luminescence regulation are influenced by radiation dose and duration, addressing limitations in X‐ray detection. Furthermore, integrating X‐ray‐induced photochromism and luminescence regulation within a single material holds great potential for applications in anti‐counterfeiting, visual imaging, medical diagnosis, and security inspection.^[^ ^10^ ^]^ This approach overcomes the limitations of interpreting single‐tone images in real scenes, enabling accurate multi‐channel diagnostics, as demonstrated in our previous studies on Ba_3_MgSi_2_O_8_: Mn^2+^ and LiAlSi_2_O_6_: Sm^3+^.^[^ ^11^ ^]^ However, challenges remain, such as the radioluminescence attenuation with non‐linear in LiAlSi_2_O_6_: Sm^3+^ and the unstable persistent luminescence signal in Ba_3_MgSi_2_O_8_: Mn^2+^, which limit their signal stability and accuracy in practical X‐ray detection. The comparison of major inorganic X‐ray detection materials is detailed in Table S2 (Supporting Information). Developing a robust X‐ray monitoring system continues to be a challenge.

**Figure 1:**
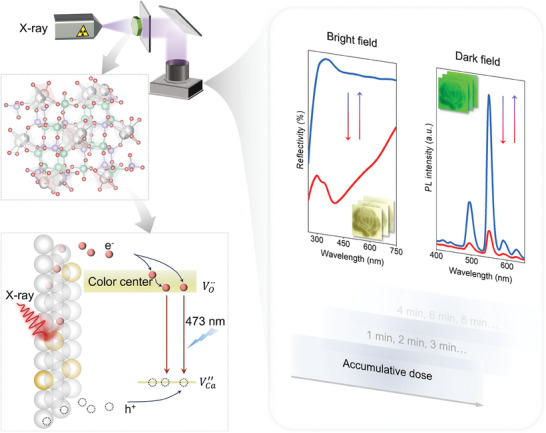
Schematic of dual‐mode X‐ray detecting and imaging in CaAl_2_Si_2_O_8_: Tb^3+^ phosphor. X‐ray‐induced oxygen vacancy‐related photochromism and luminescence modulation in the phosphor can be restored through 473 nm laser stimulation. The modulation shows a dual‐mode response in both bright and dark conditions, allowing the detection of cumulative radiation doses.

Selecting the appropriate matrix material is crucial for achieving optimal photochromic and luminescent performance. Silicate compounds like CaAl_2_Si_2_O_8_, known for their exceptional structural, thermal, and physicochemical stability, are considered suitable matrix materials.^[^ ^12^ ^]^ In this study, we selected Tb^3+^‐doped CaAl_2_Si_2_O_8_ due to its remarkable photoluminescence, radioluminescence, and X‐ray‐induced reversible photochromic properties (**Figure** **1**). Under X‐ray irradiation, we studied the degree of color change in CaAl_2_Si_2_O_8_: Tb^3+^ sintered at different temperatures, demonstrating X‐ray‐induced oxygen vacancy‐related photochromism. We studied the impact of X‐ray dose rate and exposure duration on photochromism and photoluminescence intensity, revealing a dual‐mode response in both bright and dark conditions, capable of detecting cumulative radiation doses.

The bleaching process of photochromic CaAl_2_Si_2_O_8_: Tb^3+^ under 473 nm laser stimulation was analyzed, with stability, repeatability, and fatigue resistance assessed through cyclic tests. Additionally, we identified a linear correlation between radioluminescence and X‐ray irradiation, and the radioluminescence attenuation is far below the reported LiAlSi_2_O_6_: Sm^3+^,^[^ ^11b^ ^]^ highlighting its potential for stable X‐ray imaging. A dual‐mode X‐ray detection and imaging device was then developed by combining CaAl_2_Si_2_O_8_: Tb^3+^ with PDMS, showcasing remarkable photochromism, luminescence, and radioluminescence. This material can be effectively used as ink for wearable X‐ray detection devices or processed into flexible films for imaging irregular objects. CaAl_2_Si_2_O_8_: Tb^3+^ offers a novel approach for time‐dependent cumulative X‐ray dose detection, integrating photochromism, photoluminescence, and radioluminescence into a single material for multi‐mode X‐ray detection and imaging.

## 2 Results and Discussion

**Figure 2:**
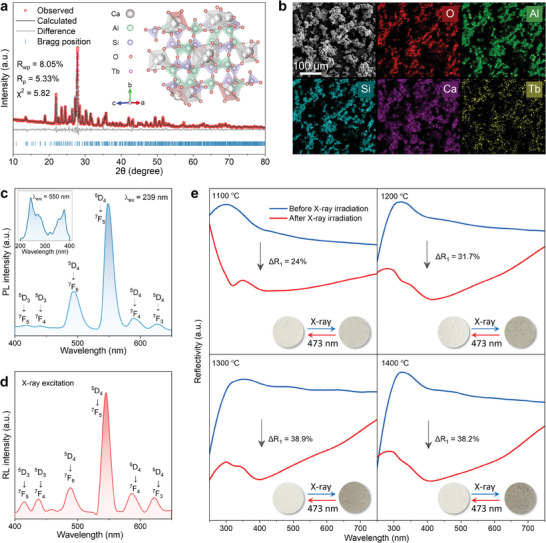
a) Rietveld refinement of the XRD profile and crystal structure for CaAl_2_Si_2_O_8_: 2 mol% Tb^3+^ sintered at 1300 °C; b) SEM and element mapping of O, Al, Si, Ga, and Tb elements; c) Emission spectra under 239 nm excitation and excitation spectra (top left) monitored at 550 nm; d) RL spectra under X‐ray exposure at a 7.4 mGy s^−1^ dose rate; e) Diffuse reflectance spectra and corresponding photographs of CaAl_2_Si_2_O_8_: 2 mol% Tb^3+^ sintered at 1100, 1200, 1300, and 1400 °C before and after X‐ray irradiation (10 min, 7.4 mGy s^−1^).

XRD and Rietveld refinement are effective for determining crystal structure parameters. The XRD patterns of CaAl_2_Si_2_O_8_ phosphors, sintered at different temperatures or doped with various concentrations of Tb^3+^, are shown in Figure S1a,b (Supporting Information). Sintering above 1300 °C resulted in high purity and crystallinity, eliminating impurities like CaCO_3_ and Al_2_O_3_ found at lower temperatures. Tb^3+^ doping did not affect phase structure, consistent with JCPDS No. 85–1660. The Rietveld refinement and corresponding cystal structure of CaAl_2_Si_2_O_8_: 2 mol% Tb^3+^ sintered at 1300 °C are depicted in **Figure** **2**a, with profile factors *R_wp_* (8.05) and *R_p_* (5.33%). The structure is triclinic with the *I‐1* space group, composed of TO_4_ tetrahedra (T = Si^4+^, Al^3+^). Ca^2+^ cations occupy large voids, forming Ca─O polyhedra with two split sites (Ca1 and Ca2), each with an occupancy factor of 0.5.^[^ ^13^ ^]^ The substitution of Tb^3+^ ions (*r* = 1.04 Å, CN = 8) for Ca^2+^ ions (r = 1.12, CN = 8) is indicated by a slightly smaller structure cell volume (V = 1338.93 Å^3^) compared to the standard unit cell volume (V_0_ of 1350.6 Å^3^).^[^ ^14^ ^]^ SEM and elements mapping confirms successful Tb^3+^ doping and uniform distribution of O, Al, Si, Ca, and Tb (Figure 2b), supported by EDS spectra (Figure S2, Supporting Information).

The photoluminescence (PL) spectra of CaAl_2_Si_2_O_8_: 2 mol% Tb^3+^ phosphor sintered at 1300 °C is shown in Figure 2c. Excitation peaks at 239 and 269 nm correspond to the 4f‐5d transitions of Tb^3+^, while additional peaks at 319, 356, and 378 nm are attributed to 4f–4f transitions within the Tb^3+^ ions’ ground state (^7^F_6_).^[^ ^15^ ^]^ Under 239 nm excitation, Tb^3+^ exhibits maximum emission at 550 nm, along with weaker peaks at 419, 441, 494, 593, and 627 nm, corresponding to the transitions of ^5^D_3_ → ^7^F_5_, ^5^D_3_ → ^7^F_4_, ^5^D_4_ → ^7^F_6_, ^5^D_4_ → ^7^F_5_, ^5^D_4_ → ^7^F_4_ and ^5^D_4_ → ^7^F_3_, respectively.^[^ ^16^ ^]^ Figure S3a (Supporting Information) shows emission spectra under different excitations (239, 254, 365, and 378 nm), with 239 nm being the optimal excitation wavelength. Emissions were also observed under commercially available UV lamps emitting at 254 and 365 nm. The PL intensity increased initially but then decreased with higher Tb^3+^ doping due to concentration quenching, with 2 mol% identified as the optimal concentration (Figure S3b, Supporting Information).^[^ ^17^ ^]^ The photoluminescence mechanism of CaAl_2_Si_2_O_8_: Tb^3+^ is illustrated in Figure S3c (Supporting Information). The radioluminescence (RL) properties of CaAl_2_Si_2_O_8_: x mol% Tb^3+^ (x = 0.25, 0.5, 1.0, 1.5, 2.0, 3.0, 4.0, and 5.0) were investigated under X‐ray exposure, showing similar emission spectra and variation trends with different Tb^3+^ doping concentrations, as presented in Figure 2d and Figure S4a (Supporting Information). Upon X‐ray excitation, the CaAl_2_Si_2_O_8_ lattice absorbs X‐rays, generating high‐energy electrons via the photoelectric effect. These electrons transition from the valence band (VB) to the conduction band (CB), creating holes in the VB. The electron‐hole pairs are then transferred to Tb^3+^ ions, ionizing them and exciting the Tb^3+^ ions to a higher energy state. As Tb^3+^ returns to its ground state, X‐ray radioluminescence is emitted. The RL mechanism is depicted in Figure S4b (Supporting Information).

The photochromic effect of CaAl_2_Si_2_O_8_: 2 mol% Tb^3+^ sintered at different temperatures (1100, 1200, 1300, or 1400 °C) was investigated under X‐ray exposure. The diffuse reflectance spectra, showing a clear absorption band from 300 to 700 nm after 10 min of X‐ray irradiation at a dose rate of 7.4 mGy s^−1^, are depicted in Figure 2e. The degree of coloration (Δ*Abs_1_*) was calculated using the equation:

(1) $$\Delta Abs_{1}=\left(R_{o\;}-R_{c}\right)/\;R_{o\;}\times \;100\%$$

where *R_o_* and *R_c_* are the diffuse reflectance intensities at 400 nm in the original and coloration states. The coloration degree increased with sintering temperature, reaching saturation at 1300 °C.

**Figure 3:**
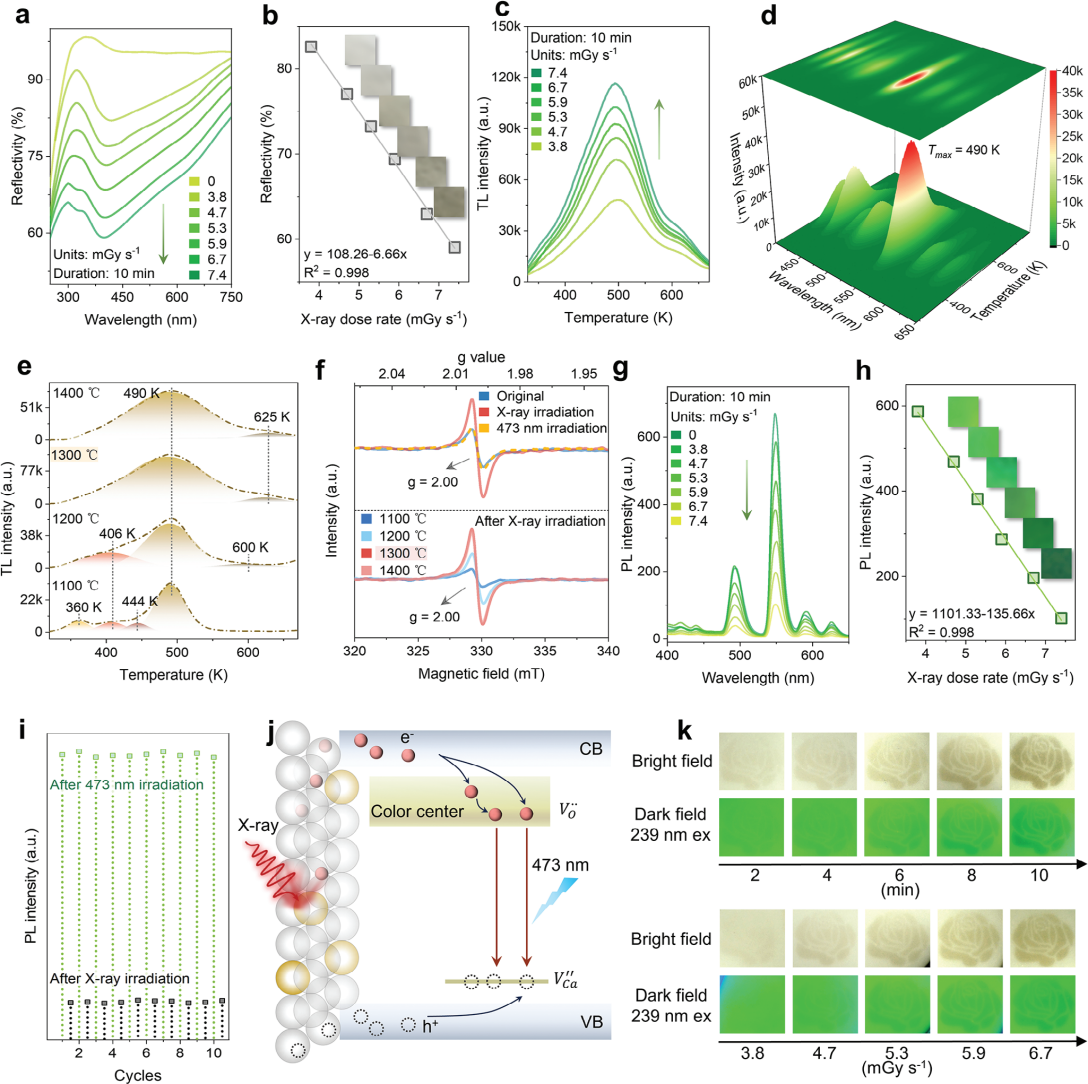
a) Diffuse reflectance spectra of CAS‐Tb under X‐ray irradiation (10 min) at different dose rate; b) Linear relationship of diffuse reflectance intensity with X‐ray dose rate with corresponding photographs; c) TL spectra of CAS‐Tb under X‐ray irradiation (10 min) at different dose rate; d) 3D‐TL spectra and contour plots of CAS‐Tb after X‐ray (7.4 mGy s^−1^, 10 min) irradiation; e) TL spectra of CAS‐Tb sintered at 1100, 1200, 1300 and 1400 °C under X‐ray irradiation (7.4 mGy s^−1^, 10 min); f) EPR spectra for original, photochromic and decoloration states in CAS‐Tb (top) and EPR spectra of CAS‐Tb under X‐ray irradiation (bottom); g) PL spectra of CAS‐Tb under X‐ray irradiation (10 min) at different dose rate; h) Linear relationship of PL intensity with X‐ray dose rate with corresponding photographs; i) 550 nm PL intensity of CAS‐Tb after alternating X‐ray (7.4 mGy s^−1^, 10 min) and 473 nm laser (30 s) irradiation cycles; j) Schematic of X‐ray‐induced photochromic mechanism; k) Rose patterns after X‐ray irradiation at different times or dose rates in bright and dark fields.

After determining the optimal sintering temperature and Tb^3+^ doping concentration, the diffuse reflectance spectra of CaAl_2_Si_2_O_8_: 2 mol% Tb^3+^ sintered at 1300 °C (referred to as CAS‐Tb) were examined under X‐ray irradiation at various dose rates for 10 min (**Figure** **3**a). As the dose rate increased (0–7.4 mGy s^−1^), the reflectance intensity decreased, with the color shifting from white to brown (photographs in Figure 3b). This intensity showed a linear relationship with the X‐ray dose rate for a given irradiation time (Figure 3b). A similar trend was observed for different exposure durations at a dose rate of 7.4 mGy s^−1^, indicating a linear relationship between reflectance intensity and cumulative X‐ray dose (Figure S5a,b, Supporting Information). These results suggest that CAS‐Tb can serve as an accurate detector and dosimeter for cumulative X‐ray doses. The bleaching of photochromic CAS‐Tb was achieved under 473 nm laser stimulation for 30 s (Figure S5c, Supporting Information) and assessed using the equation:

(2) $$\Delta Abs_{2}=R_{d\;}/R_{o\;}\times \;100\%$$

where *R_o_* and *R_d_* are the diffuse reflectance intensities at 400 nm in the original and decoloration states. Figure S5d (Supporting Information) shows a coloration degree of 39% and a decoloration degree of 99.3%, demonstrating the restorability of X‐ray‐induced photochromism in CAS‐Tb. The reusability test, shown in Figure S5e (Supporting Information), involved ten cycles of alternating X‐ray and 473 nm laser irradiation, highlighting the durability and reversibility of CAS‐Tb as an X‐ray detector and imaging device.

In inorganic phosphors, the photochromic mechanism can be attributed to phase transitions, oxygen vacancy defects, or changes in ionic valence.^[^ ^18^ ^]^ XRD and XPS analyses of Al, Si, Ca, and Tb elements in CAS‐Tb across original, colored, and decolored states (Figures S6 and S7, Supporting Information) revealed that neither phase changes nor valence state changes of the matrix and doping ions were the primary causes of photochromism. It is hypothesized that defect formation, particularly oxygen vacancies ($V_{O}^{\cdot \cdot }$), when Tb^3+^ ions are doped into the CAS host, is the main factor. High sintering temperatures lead to vacancies in Al, Si (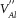 and 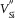), and Ca cations (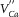), with oxygen vacancies compensating for the charge.^[^ ^19^ ^]^

Thermoluminescence (TL) analysis detects trapped charge carriers, providing insights into defect formation during X‐ray‐induced photochromism.^[^ ^20^ ^]^ TL curves obtained under X‐ray irradiation showed peaks between 350 and 650 K, indicating defects such as as $V_{O}^{\cdot \cdot }$, 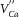, and high‐temperature‐induced 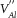 and 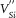 (Figure 3c; Figure S8a, Supporting Information). TL intensity increased with higher X‐ray dose rates or irradiation times, suggesting that many electrons and holes were captured by defect traps from oxygen and cation vacancies (Figure 3c; Figure S8a, Supporting Information). TL intensity gradually decreased under continuous 473 nm laser stimulation, indicating the escape and recombination of trapped carriers, leading to decoloration in the CAS‐Tb phosphor (Figure S8b, Supporting Information). These TL dynamics align with changes observed in diffuse reflectance under X‐rays and 473 nm laser irradiation. To reveal the trap natures and intrinsic mechanisms, 3D thermoluminescence (3D‐TL) spectra of CAS‐Tb after X‐ray irradiation for different durations (1, 2, and 10 min) were measured, as shown in Figure 3d and Figure S9 (Supporting Information). The 3D‐TL spectra exhibited by X‐ray (7.4 mGy s⁻¹, 10 min) exhibited characteristic emissions within 400–650 nm (from 400 to 600 K), corresponding to the Tb^3+^ emissions. The 3D‐TL intensity as a function of emission wavelength and temperature increased with longer X‐ray durations, consistent with Figure S8a (Supporting Information). These results indicated that Tb^3+^ acts as radiative recombination centers during the thermoluminescence process. To assess the impact of sintering temperature on photochromism, CAS‐Tb phosphors sintered at 1100, 1200, 1300, and 1400 °C were exposed to X‐ray irradiation (7.4 mGy s^−1^, 10 min) (Figure 3e; Figure S10, Supporting Information). The TL curves were fitted with Gaussian peaks, and the corresponding defect trap depths were estimated using the equation:^[^ ^21^ ^]^

(3) $$E=T_{m}\;/\;500$$

where *E* (eV) is the defect depth and *T_m_* (K) is the temperature of the TL spectra. The TL curves were fitted with Gaussian peaks, and the corresponding defect trap depths were estimated using the equation (Table S3, Supporting Information), with shallow traps in samples sintered at 1100 and 1200 °C and deeper traps in those sintered at 1300 and 1400 °C. TL intensity increased with sintering temperatures up to 1300 °C, suggesting that higher temperatures enhance both the quantity and depth of defects, which likely improves photochromic performance.

To confirm the presence of oxygen vacancies, the EPR spectrum of CAS‐Tb phosphor was analyzed in its original, colored, and decolored states, as shown at the top of Figure 3f. The EPR signal at *g* = 2.00, attributed to paramagnetic single‐ionized oxygen vacancies ($V_{O}^{\cdot }$), indicates vacancy defects associated with unpaired free electrons.^[^ ^22^ ^]^ The EPR intensity increased after X‐ray irradiation (7.4 mGy s⁻¹, 10 min) and returned to its original level after 473 nm laser treatment (30 s), reflecting the increase and decrease in oxygen vacancies during the photochromism and decoloration processes. Additionally, as shown at the bottom of Figure 3f, the EPR intensity increased with higher sintering temperatures, peaking at 1300 °C, similar to the trend seen in TL intensity. This response is key to understanding CAS‐Tb's photochromic behavior and explains why higher sintering temperatures enhance photochromism, as seen in Figure 2e.

The photochromism and decoloration mechanism in CAS‐Tb, illustrated in Figure 3j, involves the excitation of electrons from the valence band (VB) to the conduction band (CB) under X‐ray irradiation, creating holes in the VB. The excited electrons and holes are captured by $V_{O}^{\cdot \cdot }$ and cation vacancies (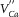, 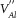 and 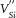), leading to the formation of color centers and resulting in coloration. Exposure to 473 nm light provides trapped electrons with enough energy to escape the defects and recombine with the holes, causing the color centers to fade.

The PL intensity of CAS‐Tb decreased under X‐ray irradiation as the dose rate or exposure time increased, as shown in Figure 3g and Figure S11a (Supporting Information). This attenuation demonstrated a linear relationship with X‐ray dose rates ranging from 0.38 to 7.4 mGy s⁻¹ over 10 min, modulating the Tb^3+^ luminescence from bright green to dark green (Figure 3h and inset). A linear correlation between PL intensity and X‐ray irradiation duration (7.4 mGy s⁻¹) was also observed (Figure S11b, Supporting Information), enabling the conversion of irradiation time into accumulated X‐ray dose. Under continuous 473 nm laser stimulation for up to 30 s, the photochromic CAS‐Tb recovered its PL intensity to its original level (Figure S11c, Supporting Information). The modulation degree (Δ*PL_1_*) and recovery degree (Δ*PL_2_*) were calculated using the following equations:

(4) $$\Delta PL_{1}=\left(PL_{o}\;-PL_{c}\right)\;/PL_{o}\;\times \;100\%$$

(5) $$\Delta PL_{2\;}=PL_{d\;}/PL_{o}\;\times \;100\%$$

where *PL_o_*, *PL_c_*, and *PL_d_* are the PL intensities at 550 nm in the original, colored, and decolored states. Under X‐ray irradiation at 7.4 mGy s^−1^ and 473 nm laser irradiation, CAS‐Tb exhibited a modulation degree (Δ*PL_1_*) of 84.7% and a recovery degree (Δ*PL_2_*) of 99.9%, as shown in Figure S12 (Supporting Information). Even after ten cycles of alternating X‐ray irradiation (7.4 mGy s⁻¹ for 10 min) and 473 nm laser irradiation (30 s), CAS‐Tb maintained stable modulation and recovery of PL intensity (Figure 3i). Rose patterns obtained via mask‐covered methods after prolonged X‐ray exposure at various dose rates or times exhibited distinct photochromic and PL modulation degrees (Figure 3k). The varying color depths under different irradiation conditions illustrate reversible luminescence modulation based on X‐ray‐induced photochromism, demonstrating its potential for multi‐functional anti‐counterfeiting and visual detection in both dark and bright field conditions.

Figure S13 (Supporting Information) shows the PL decay curves of CAS‐Tb in its original and photochromic states. Under 239 nm excitation, the PL peak at 550 nm had lifetimes of 3.93 and 3.15 µs for the original and photochromic samples, respectively. The slight decrease after X‐ray exposure suggests minimal resonance energy transfer from luminescence to color centers. During X‐ray irradiation, color centers formed in CAS‐Tb by capturing excited electrons and holes, creating an absorption band (300–700 nm) that overlaps with the Tb^3+^ emission band (400–650 nm). Upon 473 nm laser stimulation, these color centers bleach, reducing absorption and restoring PL. This indicates that luminescence can be dynamically modulated by the photochromic effect through the overlap of absorption and emission spectra.

**Figure 4:**
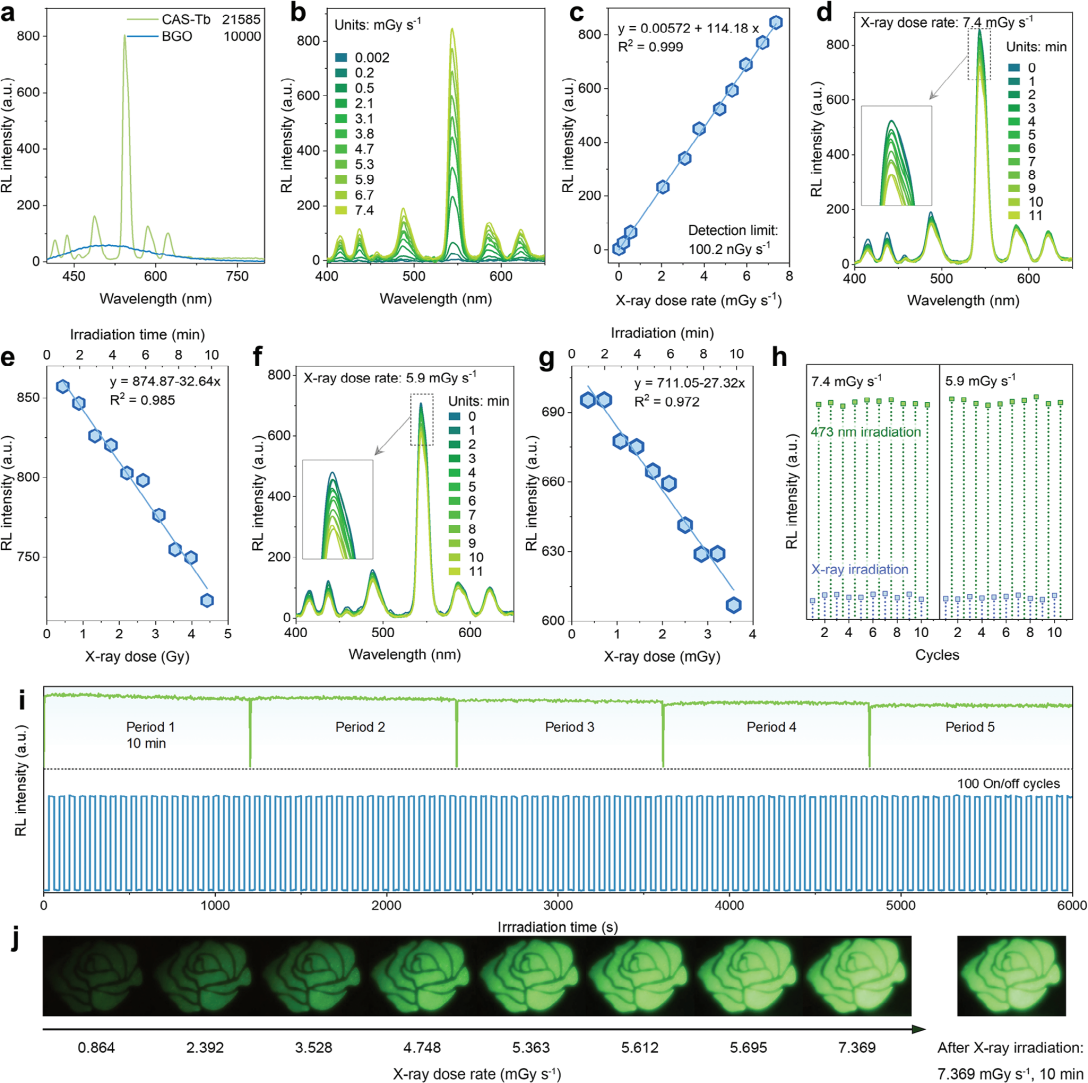
a) RL spectrum of BGO and CAS‐Tb; b) RL spectrum of CAS‐Tb under X‐ray exposure at different dose rates; c) Linear correlation between RL intensity and X‐ray dose rate; d) RL spectrum of CAS‐Tb under different irradiation time at 7.4 mGy s^−1^; e) Linear correlation between RL intensity and time‐dependent X‐ray dose at 7.4 mGy s^−1^; f) RL spectrum of CAS‐Tb under different irradiation time at 5.9 mGy s^−1^; g) Linear correlation between RL intensity at 544 nm peak and time‐dependent X‐ray dose at 5.9 mGy s^−1^; h) 544 nm RL intensity of CAS‐Tb after alternating X‐ray and 473 nm laser irradiation cycles; i) Photostability of CAS‐Tb: RL at 544 nm was recorded under continuous X‐ray irradiation for 10 min (top) and 100 on‐off cycles of X‐ray exposure with 30 s time interval (bottom); j) Photographs of “rose” patterns formed by mask‐covered CAS‐Tb under various X‐ray dose rates and 10 min duration.

The RL of CAS‐Tb is highly sensitive to X‐ray exposure. The RL light yield of CAS‐Tb was measured using the commercial scintillator BGO crystal as a reference, with a light yield of 10000 photons MeV^−1^. CAS‐Tb exhibited a light yield of 21585 photons MeV^−1^, demonstrating 2.16 times higher X‐ray sensitivity than BGO (**Figure** **4**a). The RL intensity of CAS‐Tb increased linearly under X‐ray exposure with dose rates ranging from 0.003 to 7.4 mGy s^−1^. The detection limit, derived from the linear relationship between RL intensity and X‐ray dose rate, was determined to be 100.2 nGy s^−1^ (Figure 4b,c). The RL spectra were also recorded for X‐ray exposures up to 10 min at dose rates of 7.4 and 5.9 mGy s⁻¹ (Figure 4d,f). The RL reduction (Δ*RL*) can be calculated using the equation:

(6) $$\Delta RL=\left(RL_{o\;}-RL_{c}\right)\;/\;RL_{o}\;\times \;100\%$$

where *RL_o_*, *RL_c_*, and *RL_d_* are the RL intensities at 554 nm before and after X‐ray exposure. Only a slight reduction (Δ*RL*) in RL intensity of 14.8% and 14.4% was observed under 10 min of X‐ray irradiation at 7.4 and 5.9 mGy s⁻¹, respectively. Additionally, the RL intensity exhibited a linear correlation with X‐ray irradiation time (Figure 4e,g), offering an accurate dose assessment and high imaging quality compared to the published research. The slightly reduced RL intensity can be recovered through 473 nm laser stimulation, as shown in the cyclic characterization under alternating X‐ray irradiation (7.4/5.9 mGy s^−1^ for 10 min) and 473 nm laser irradiation (30 s) (Figure 4h; Figure S14, Supporting Information). To verify photostability, we measured the changes in RL intensity under continuous X‐ray irradiation and during on‐off X‐ray cycles (Figure 4i). CAS‐Tb demonstrated high photostability, maintaining consistent RL intensity during continuous X‐ray irradiation for 10 min across 5 periods and over 100 on‐off X‐ray exposure cycles with 30 s intervals. The results in Figure 4j showcase “rose” patterns formed by mask‐covered CAS‐Tb materials using various X‐ray dose rates. Notably, even after 10 min of exposure at 7.4 mGy s^−1^, no significant reduction in brightness was observed, highlighting CAS‐Tb's potential for reusability and stability in visual X‐ray imaging.

**Figure 5:**
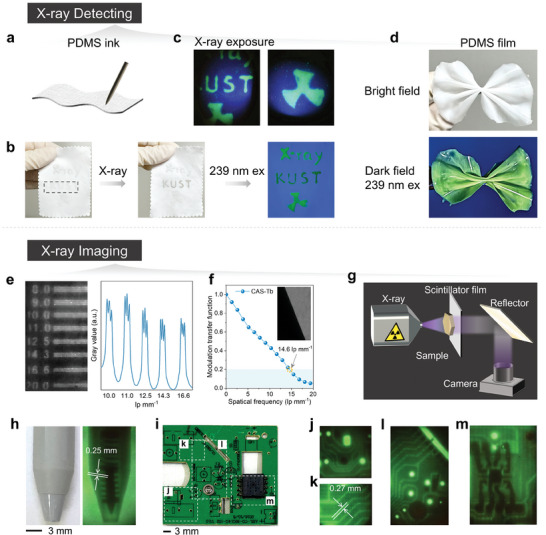
a) Schematic of fabric with CAS‐Tb handwriting ink; b) Patterns on fabric before and after X‐ray irradiation at 7.4 mGy s^−1^ for 10 min; c) Patterns on fabric under X‐ray exposure at 7.4 mGy s^−1^; d) Flexible CAS‐Tb film in bright and dark field; e) X‐ray imaging of a standard line pair card (left) and the gray value modulation (right); f) MTF of CAS‐Tb film measured using the slanted‐edge method; g) Schematic of the X‐ray imaging system; Detailed X‐ray imaging of the ballpoint pen h) and circuit board i–m).

Based on the previous discussion, a proof‐of‐concept PDMS ink embedded with CAS‐Tb phosphor was developed for handwriting various labels on fabric, such as “X‐ray,” “KUST,” and radio‐labeling patterns (**Figure** **5**a). These patterns were used to test the dual‐mode X‐ray detection capabilities by integrating photochromism and luminescence modulation in CAS‐Tb PDMS ink. When irradiated with X‐rays at 7.4 mGy s^−1^ for 10 min, the “KUST” label changed color from white to brown in the bright field and showed reduced green luminescence under 239 nm excitation in the dark field (Figure 5b). All patterns displayed green radioluminescence (RL) under X‐ray exposure at 7.4 mGy s^−1^ (Figure 5c), demonstrating potential applications in wearable X‐ray imaging and multi‐mode anti‐counterfeiting labels. The variations in color depth under different lighting conditions highlight CAS‐Tb's suitability for high‐resolution, multifunctional X‐ray detection.

The PDMS ink containing CAS‐Tb phosphor was also molded into a flexible film that emitted green luminescence under 239 nm excitation in a dark field (Figure 5d). The CAS‐Tb film demonstrated a superior spatial resolution of 14.3 lp mm⁻¹ using a standard line pair card (Figure 5e). To further quantify the resolution, the slanted‐edge method was applied to X‐ray images of aluminum sheets (thickness ≈1 mm), producing an MTF curve. The spatial resolution (MTF = 0.2) of CAS‐Tb was measured at 14.6 lp mm^−1^, aligning with the observed limit in Figure 5f. This result confirms the high quality of X‐ray imaging achieved with CAS‐Tb. An X‐ray imaging setup was designed, with the sample placed between the X‐ray source and the CAS‐Tb film, and images were captured using a standard camera (Figure 5g). The CAS‐Tb X‐ray imager effectively provided detailed structural information of objects like a ballpoint pen and a circuit board under X‐ray exposure at 7.4 mGy s^−1^ (Figure 5h,i). The magnified imaging of the circuit board's marked area is shown in Figure 5j–m. These photochromic and luminescent properties of CAS‐Tb in both handwriting ink and flexible film significantly enhance X‐ray signal capture, advancing the technology for detecting and imaging diverse objects beyond traditional flat‐panel detectors.

## 3 Conclusion

In this work, Tb^3+^ doped CaAl_2_Si_2_O_8_ phosphor was developed, exhibiting X‐ray‐induced photochromism, photoluminescence, and radioluminescence for dual‐mode X‐ray detection and imaging. Under X‐ray irradiation, oxygen vacancy‐related color centers caused a color shift from white to brown and reduced green luminescence intensity. The coloration and luminescence of CaAl_2_Si_2_O_8_: Tb^3+^ can be adjusted by varying the X‐ray dose rate and duration, enabling cumulative X‐ray dose detection. The effects are reversible through 473 nm laser stimulation. PDMS ink and flexible films based on CaAl_2_Si_2_O_8_: Tb^3+^ were designed, demonstrating potential applications in wearable X‐ray detection and imaging devices for multi‐mode displays, environmental monitoring, industrial safety, and anti‐counterfeiting.

## Conflict of Interest

The authors declare no conflict of interest.

## Author Contributions

Z.Y. conceived and supervised the project. X.B. conducted the experiments with assistance from J.L. J.L. and X.B. prepared the figures. Z.Y., J.L., and X.B. wrote and revised the manuscript. All the authors discussed the results and feedback on the manuscript.

## Supporting information



Supporting Information

## Data Availability

The data that support the findings of this study are available in the supplementary material of this article.
