# Supplementary material for: Flexible X‐Ray Detector for Cumulative Dose Monitoring Through Reversible Photochromism and Luminescence Modulation
Source: Adv Sci (Weinh). 2024 Dec 23;12(6):2412986. doi: 10.1002/advs.202412986 (PMC11809380; doi:10.1002/advs.202412986)
Supplement: Supplementary file 1 — Supporting Information [file ADVS-12-2412986-s001.docx]

Supporting Information

**Flexible X-Ray Detector for Cumulative Dose Monitoring Through Reversible Photochromism and Luminescence Modulation**

*Xue Bai, Yueteng Zhang,* *Heping Zhao, Yingzhu Zi, Zan Xu, Anjun Huang, Yangke Cun, Yue Liu, Zhiguo Song,* *Jianbei Qiu, Jiayan Liao* and Zhengwen Yang**

*X. Bai, Y. Zhang, H. Zhao, Y. Zi, Z. Xu, A. Huang, Y. Cun, Y. Liu, Z. Song, J. Qiu, Z. Yang*

*College of Materials Science and Engineering, Kunming University of Science and Technology, Kunming, 650093, China*

*E-mail: yangzw@kust.edu.cn*

*H. Zhao, Y. Zi, Z. Yang*

*Southwest United Graduate School, Kunming 650092, China*

*J. Liao*

*Institute for Biomedical Materials and Devices (IBMD), Faculty of Science, University of Technology Sydney, NSW 2007, Australia*

*E-mail: jiayan.liao@uts.edu.au*

This file includes Experimental Section, Table S1 to Table S3 and Figures S1 to Figure S14.

**Experimental Section**

***Phosphor sample preparation*:**

The raw materials, CaCO_3_ (99.99%), Al_2_O_3_ (99.99%), SiO_2_ (99.99%) and Tb_2_O_3_ (99.99%) were used to synthesize the CaAl_2_Si_2_O_8_: Tb^3+^ phosphor via a high-temperature solid-state reaction method. The required raw materials were weighted according to stoichiometric ratio for the selected system CaAl_2_Si_2_O_8_: x mol% Tb^3+^ (x = 0, 0.25, 0.5, 1, 1.5, 2, 3, 4, 5), and mixed with alcohol as a dispersant in an agate mortar. Then the mixtures were sintered at different temperatures (1100, 1200, 1300 and 1400 ℃ for 5 h) in an air atmosphere with a heating rate of 4.5 ℃ min^-1^. After cooling to room temperature, the phosphors were finely ground for subsequent characterization.

***Flexible film preparation*:**

The re-ground CaAl_2_Si_2_O_8_: Tb^3+^ phosphors were then mixed with polydimethylsiloxane (PDMS) substrates and curing agent with a weight ratio of 5:10:1. The composite was then poured into a glass mold or manually applied onto the fabric. After heated at 70 ℃ for 4 h, the flexible CaAl_2_Si_2_O_8_: Tb^3+^ film and handwritten pattern were obtained.

***Characterization*:**

The crystallinity and phase structure of the phosphors were analyzed by X-ray diffractometer (XRD, Rigaku Ultima IV). Morphology and elemental distribution were examined with scanning electron microscopy (SEM, ZEISS Sigma 300) equipped with Energy dispersive spectrum (EDS). The UV-VIS spectrophotometer (Hitachi U-4100) was used to record diffuse reflectance spectra. The fluorescence spectrophotometer (Hitachi F-7000) with a 150 W xenon lamp was employed for measuring photoluminescence (PL) spectra, the corresponding PL decay curves were obtained using the fluorescence spectrometer (Edinburgh FLS1000). Radioluminescence (RL) spectra were measured using a D8 Focus diffractometer (Bruker) with Cu K*α* radiation (*λ* = 0.15405 nm) and a FLAME-S-XR1-ES spectrophotometer (Ocean Optics USB2000 + XR1-ES). X-ray photoelectron spectroscopy (XPS, Therma ESCALAB 250XI) was used to analyze the valence states of the constituent elements under Al-K*α* laser irradiation in a vacuum. The electron paramagnetic resonance (EPR) spectra were obtained using a JEOL JES-FA200 spectrometer at 9.2 GHz and 150 K. The thermoluminescence (TL) spectra were measured with a thermoluminescence meter (FJ-427A1) at a heating rate of 1 °C s^-1^.

***Calculation of Modulation Transfer Function:***

Modulation transfer function (MTF) determines the spatial resolution of an imaging system and represents its ability to transfer input signal modulation of spatial frequency relative to its output. MTF is calculated as the ratio of the output contrast to the input contrast of the image. As the contrast of the output image consistently remained lower than that of the input image, the resulting MTF value ranged between 0 and 1. The spatial resolution can be determined by the corresponding spatial frequency when the MTF value decreases to 0.2. The slanted-edge method was employed to calculate the MTF curve in this study. The X-ray image was acquired using a piece of Aluminum sheets (thickness: ≈1 mm) with sharp edge, allowing for the edge spread function (ESF) through analysis of the slanted-edge profile of this image. The MTF can be calculated by the following equation: ^[1]^

$$MTF\left( \nu\right)=F \left( LSF\left( x \right) \right)=F\frac{dESF(x)}{dx}$$

Where $\nu$ is the spatial frequency, $x$ is the position of pixels, the line spread function (LSF) is the derivative of ESF and the MTF was the Fourier transform of LSF. The MTF calculation on image was operated through software “Image J”.

***Reversible coloration and luminescence imaging*:**

X-ray-induced coloration was studied using a D8 focusing diffractometer (Bruker) with Cu-K*α* radiation (*λ* = 0.15405 nm) and a small X-ray tube (Amptek). Bleaching process was induced using a 473 nm laser (MBL-N-473-AOM) at a power density of 186.84 W cm^-2^. Photochromic and luminescence images were captured with a complementary metal-oxide-semiconductor (CMOS) sensor camera (Nikon D850). The taking paraments of X-ray imaging photographs were shown as follow:

**Table S1**. Taking paraments of photochromic, photoluminescence, radioluminescence and X-ray imaging photographs.

| Camera mode: NIKON D850 | | | |
| --- | --- | --- | --- |
| Mode | **Aperture value** | **Exposure time** | **ISO speed** |
| Photochromic | f/3.2 | 1/80 s | ISO-1250 |
| Photoluminescence | f/3.2 | 1/4 s | ISO-1250 |
| Radioluminescence | f/3.2 | 5 s | ISO-1000 |
| X-ray imaging | f/3.3 | 10 s | ISO-800 |

**Table S2.** The comparison of this work and major inorganic X-ray detection materials.

| Materials | | Detection mode | Signal stability | Real-time detection | Cumulative detection | Flexible imaging |
| --- | --- | --- | --- | --- | --- | --- |
| Photochromic phosphors | **This work** | **PC, PL, RL** | **√** | **√** | **√** | **√** |
|  | LiAl_2_Si_2_O_6_:Sm ^[2]^ | PC, PL, RL | RL × | √ | √ | √ |
|  | Ba_2_SiO_4_:Eu ^[3]^ | PC, PL | √ | √ | √ | √ |
|  | Ba_3_MgSi_2_O_8_:Mn^2+ [4]^ | PC, PL, PersRL | PersRL × | √ | √ | √ |
| Metal halide perovskites ^[5]^ | | RL | √ | √ | × | √ |
| Persistent luminescence nanomaterials ^[6]^ | | PersRL | × | √ | √ | √ |

**Table S3.** TL peaks and defect trap energy in CAS-Tb phosphors sintered at 1100, 1200, 1300 and 1400 ℃.

| Sample | 1100 ℃ | | | | 1200 ℃ | | | 1300℃ | | 1400 ℃ | |
| --- | --- | --- | --- | --- | --- | --- | --- | --- | --- | --- | --- |
| Peak | 1 | 2 | 3 | 4 | 1 | 2 | 3 | 1 | 2 | 1 | 2 |
| *T_m_*/K | 360 | 406 | 444 | 490 | 406 | 490 | 600 | 490 | 625 | 490 | 625 |
| *E*/eV | 0.72 | 0.812 | 0.888 | 0.98 | 0.812 | 0.98 | 1.2 | 0.98 | 1.25 | 0.98 | 1.25 |


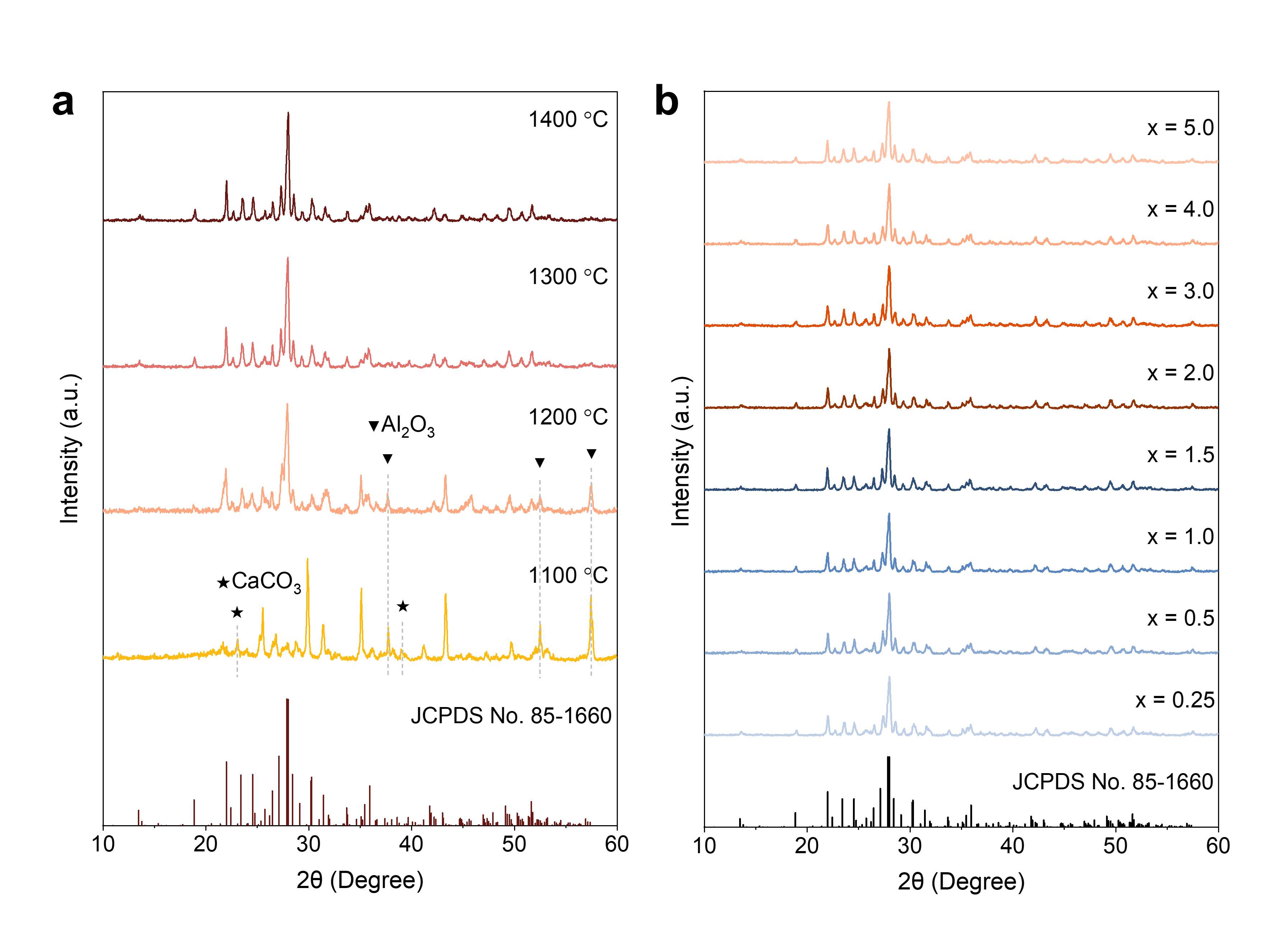


**Figure S1. a)** XRD patterns of CaAl_2_Si_2_O_8_: 2 mol% Tb^3+^ phosphors sintered at 1100, 1200, 1300 and 1400 ℃; **b)** XRD patterns of CaAl_2_Si_2_O_8_: x mol% Tb^3+^ (x = 0.25, 0.5, 1.0, 1.5, 2.0, 3.0, 4.0, 5.0) phosphors sintered at 1300 ℃.


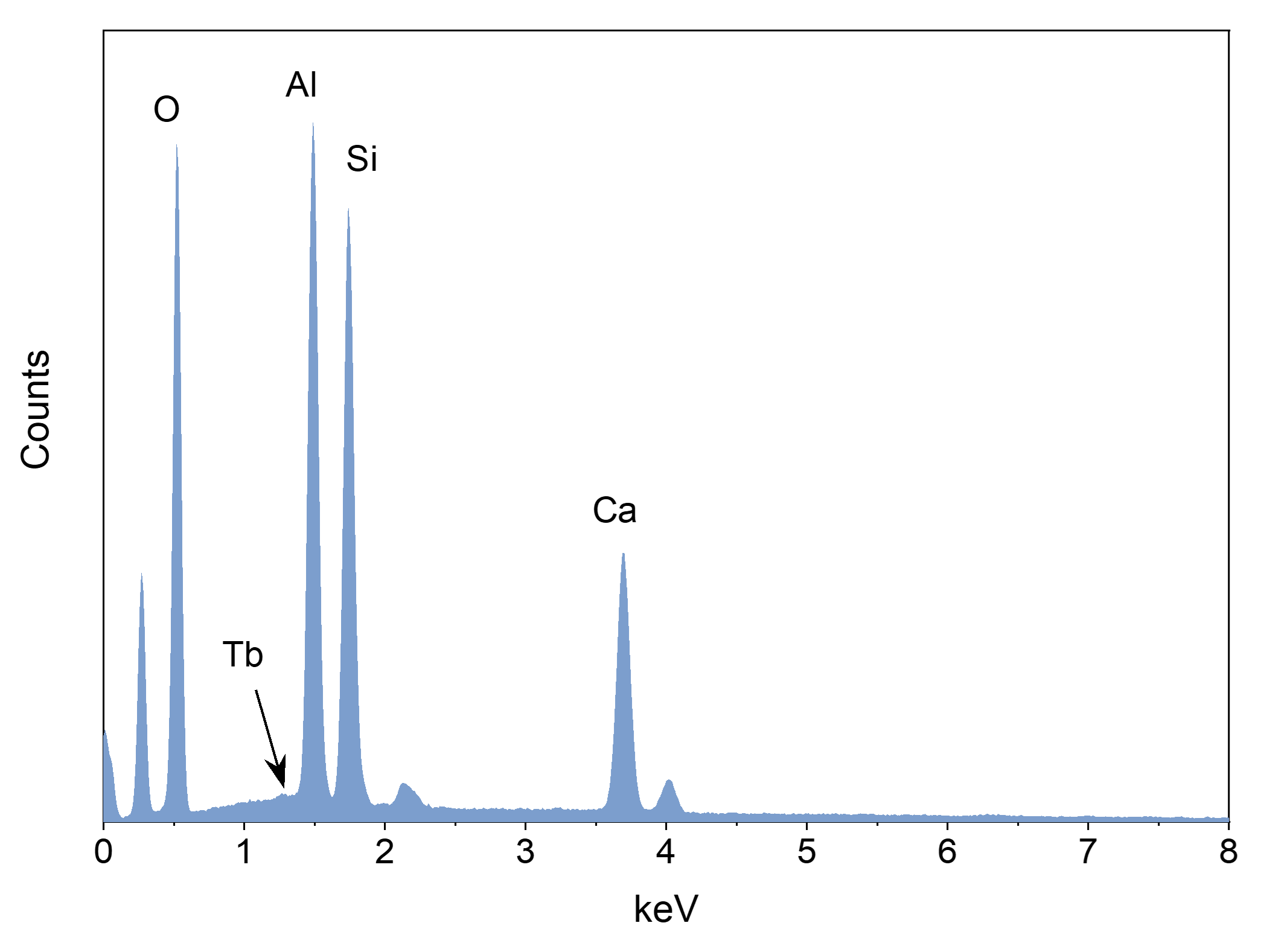


**Figure S2.** EDS spectrum of CaAl_2_Si_2_O_8_: 2 mol% Tb^3+^ phosphor sintered at 1300 ℃.


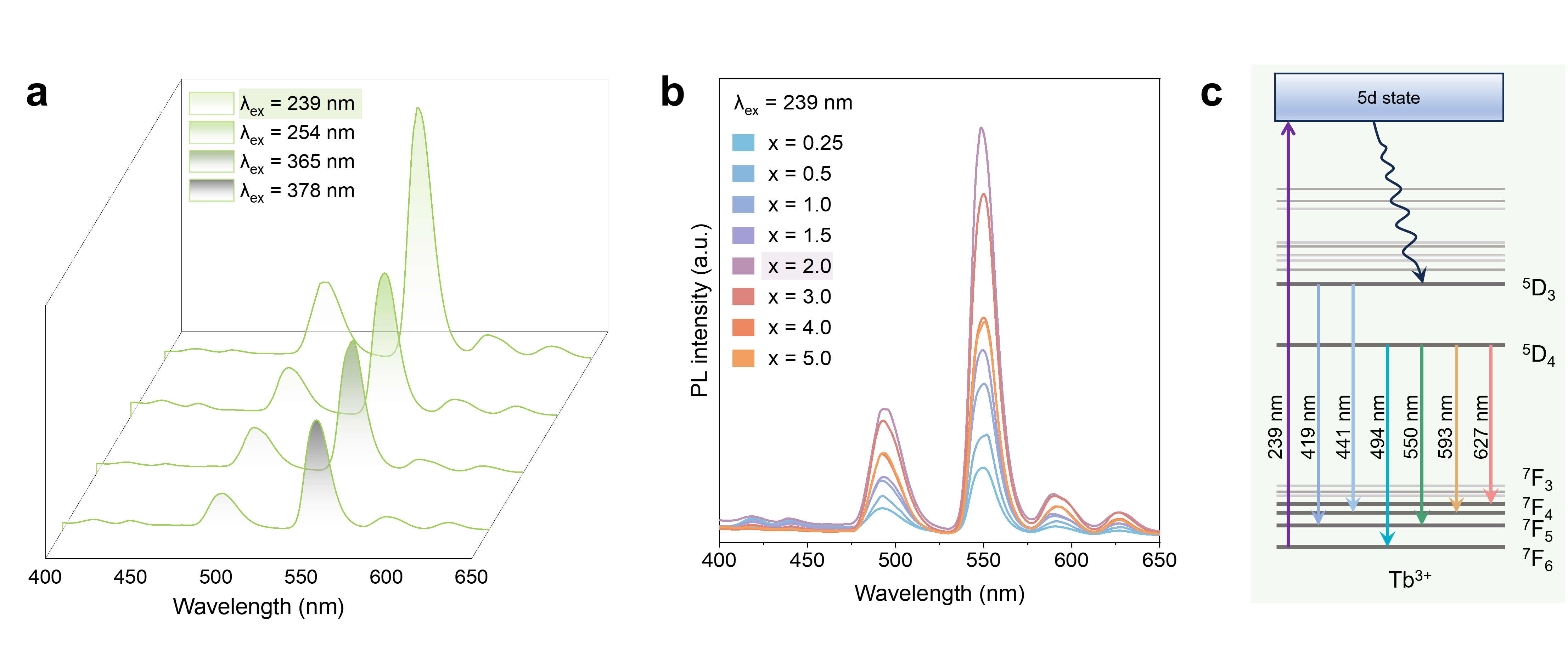


**Figure S3. a)** Photoluminescence spectrum of CaAl_2_Si_2_O_8_: 2 mol% Tb^3+^ phosphor sintered at 1300 ℃ under 239, 254, 365 and 378 nm excitation; **b)** Photoluminescence spectrum of CaAl_2_Si_2_O_8_: x mol% Tb^3+^ (x = 0.25, 0.5, 1.0, 1.5, 2.0, 3.0, 4.0, 5.0) phosphors sintered at 1300 ℃; **c)** Photoluminescence mechanism of CaAl_2_Si_2_O_8_: Tb^3+^.


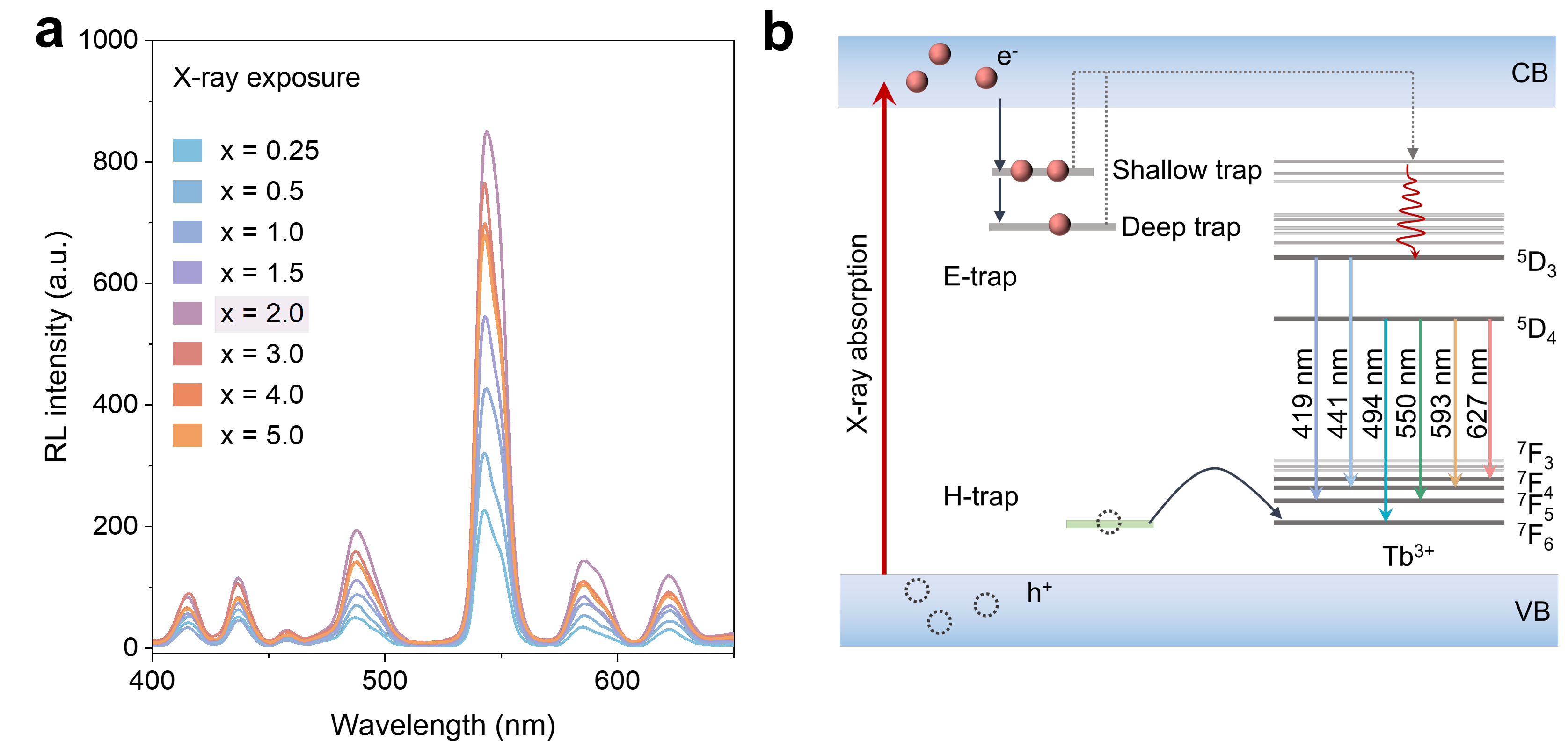


**Figure S4. a)** Radioluminescence (RL) properties of CaAl_2_Si_2_O_8_: x mol% Tb^3+^ (x = 0.25, 0.5, 1.0, 1.5, 2.0, 3.0, 4.0, 5.0); **b)** radioluminescence mechanism of CaAl_2_Si_2_O_8_: Tb^3+^ under X-ray exposure.


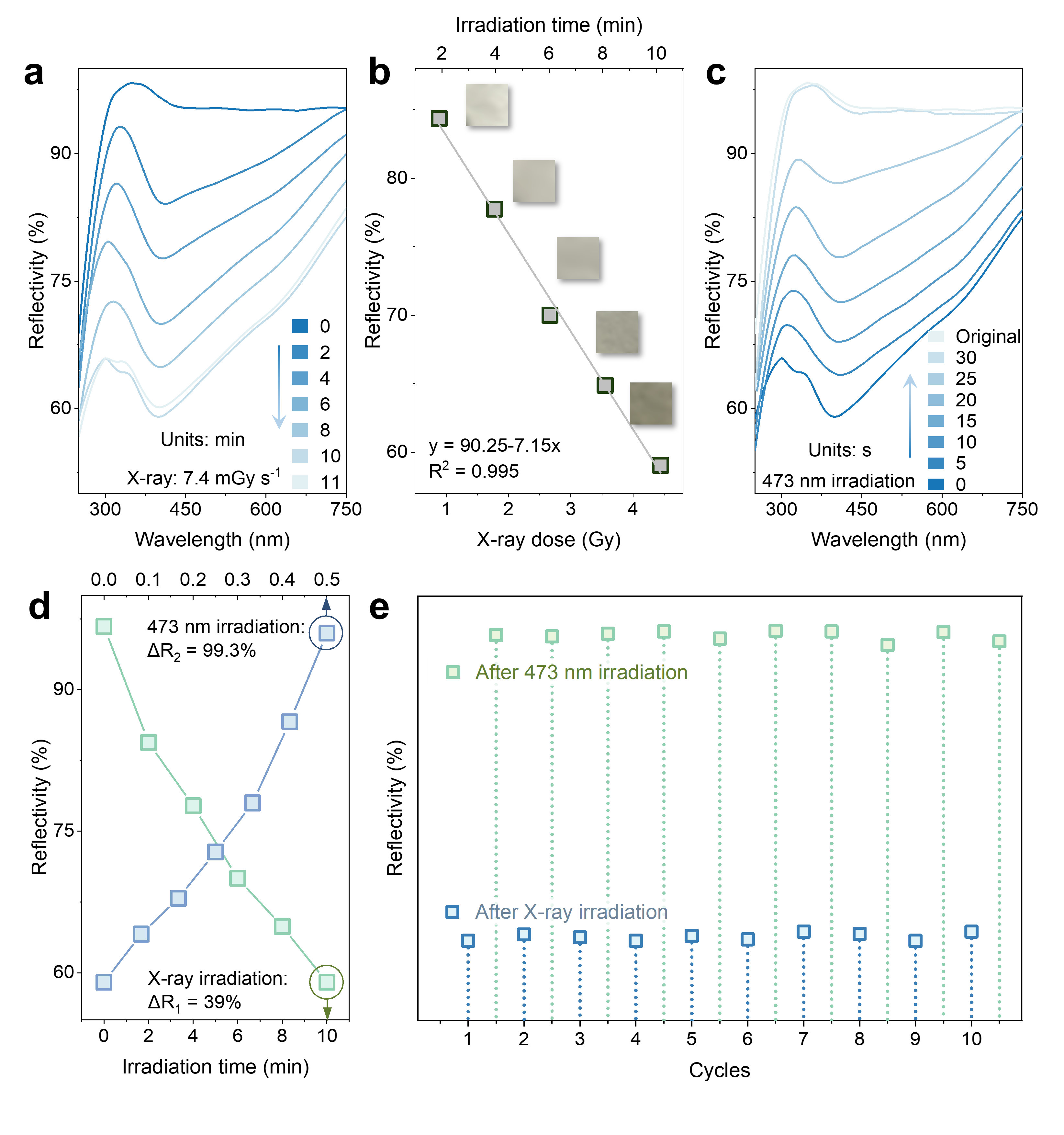


**Figure S5. a)** Diffuse reflectance spectra of CAS-Tb under X-ray irradiation (7.4 mGy s^-1^) for different durations; **b)** Linear relationship between diffuse reflectance intensity and X-ray irradiation time; **c)** Diffuse reflectance spectra of CAS-Tb under 473 nm irradiation for different durations; **d)** Diffuse reflectance intensity at 400 nm under X-ray (7.4 mGy s^-1^) and 473 nm laser irradiation for different times; **e)** 400 nm diffuse reflectance intensity of CAS-Tb after ten cycles of alternating X-ray (7.4 mGy s^-1^, 10 min) and 473 nm laser (30 s) irradiation.


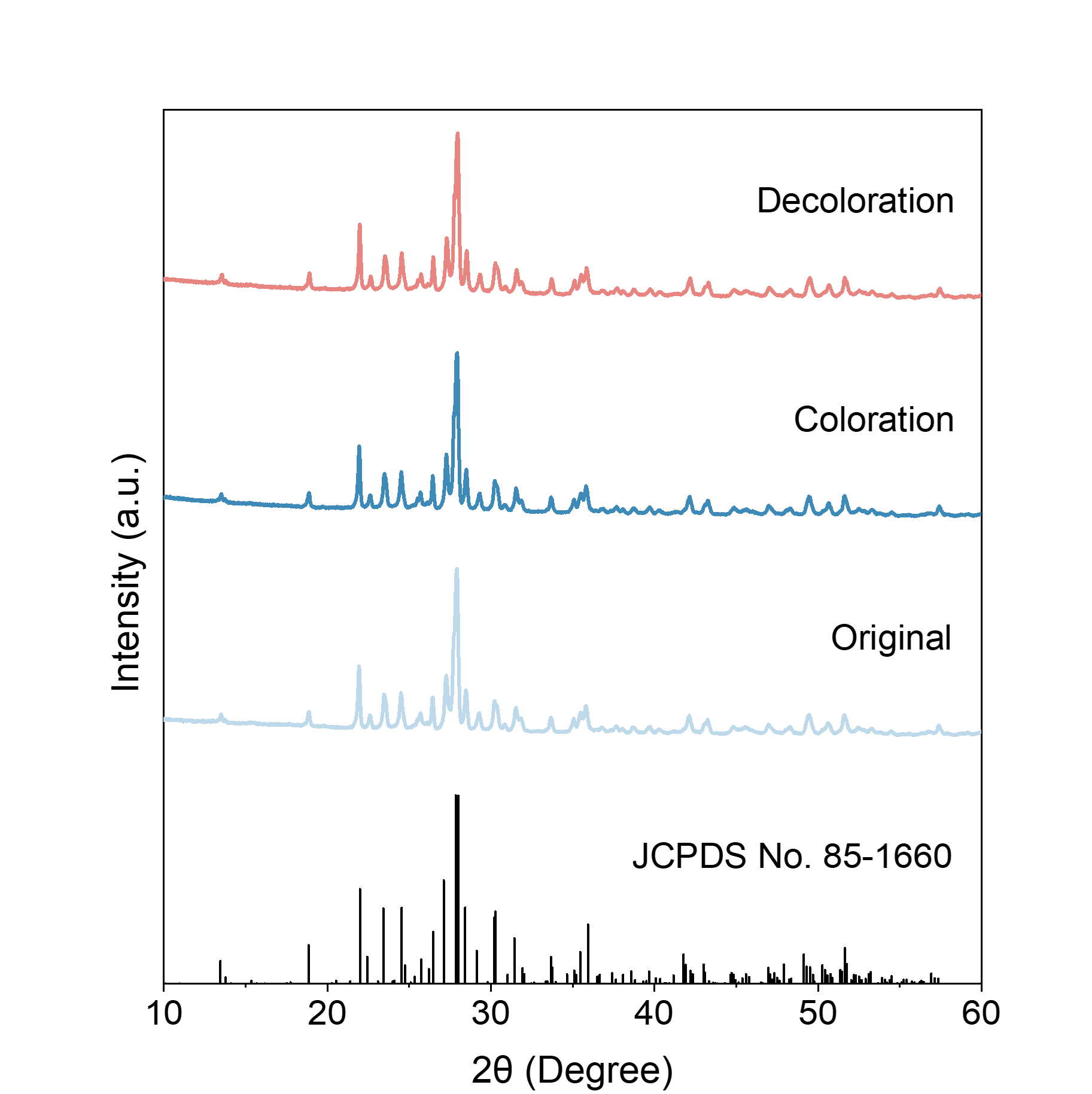


**Figure S6.** XRD patterns of CAS-Tb in its original, colored, and decolored states.


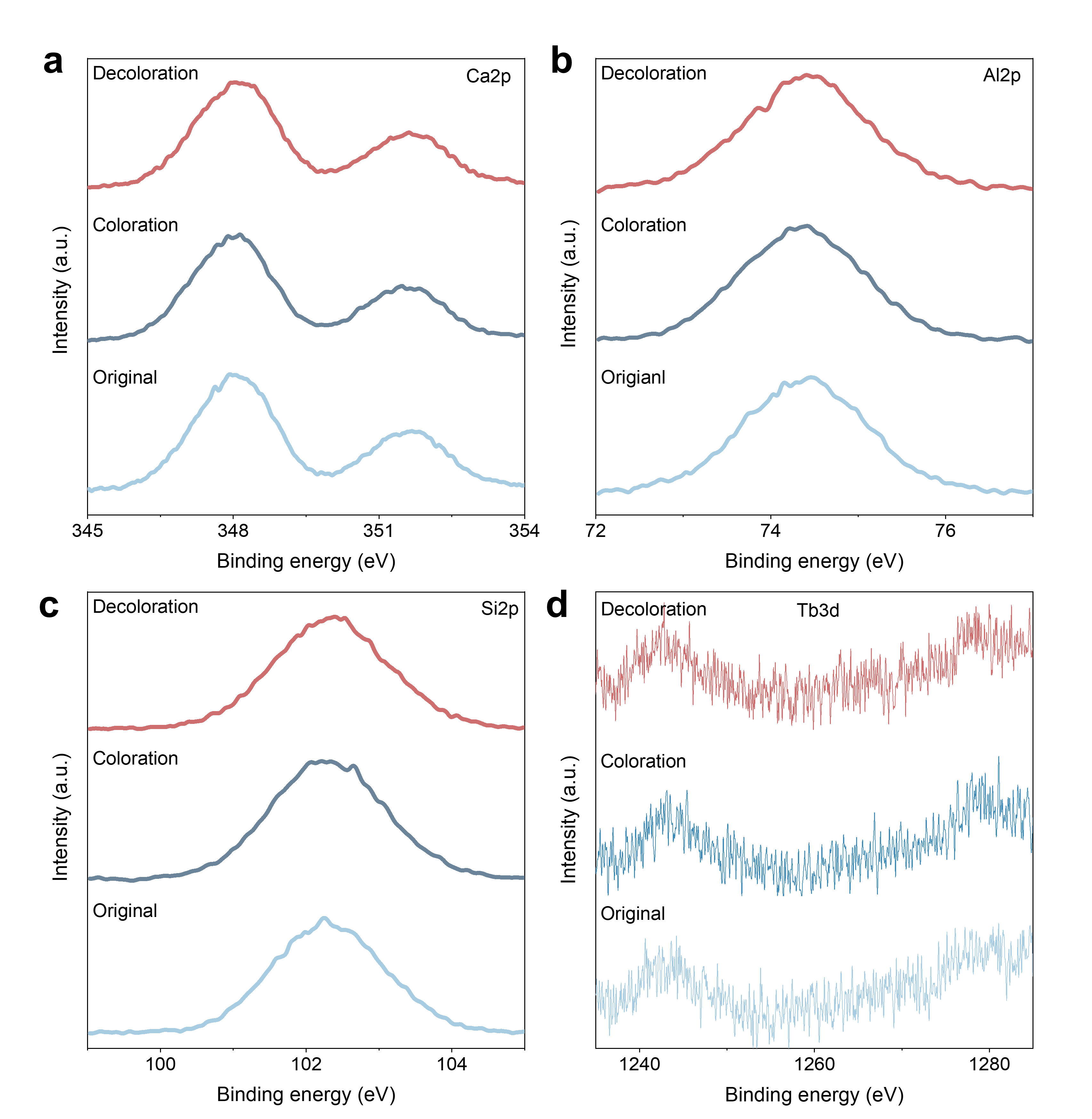


**Figure S7.** XPS spectrum of Ca **a)**, Al **b)**, Si **c)** and Tb **d)** elements in CAS-Tb in its original, colored, and decolored states.


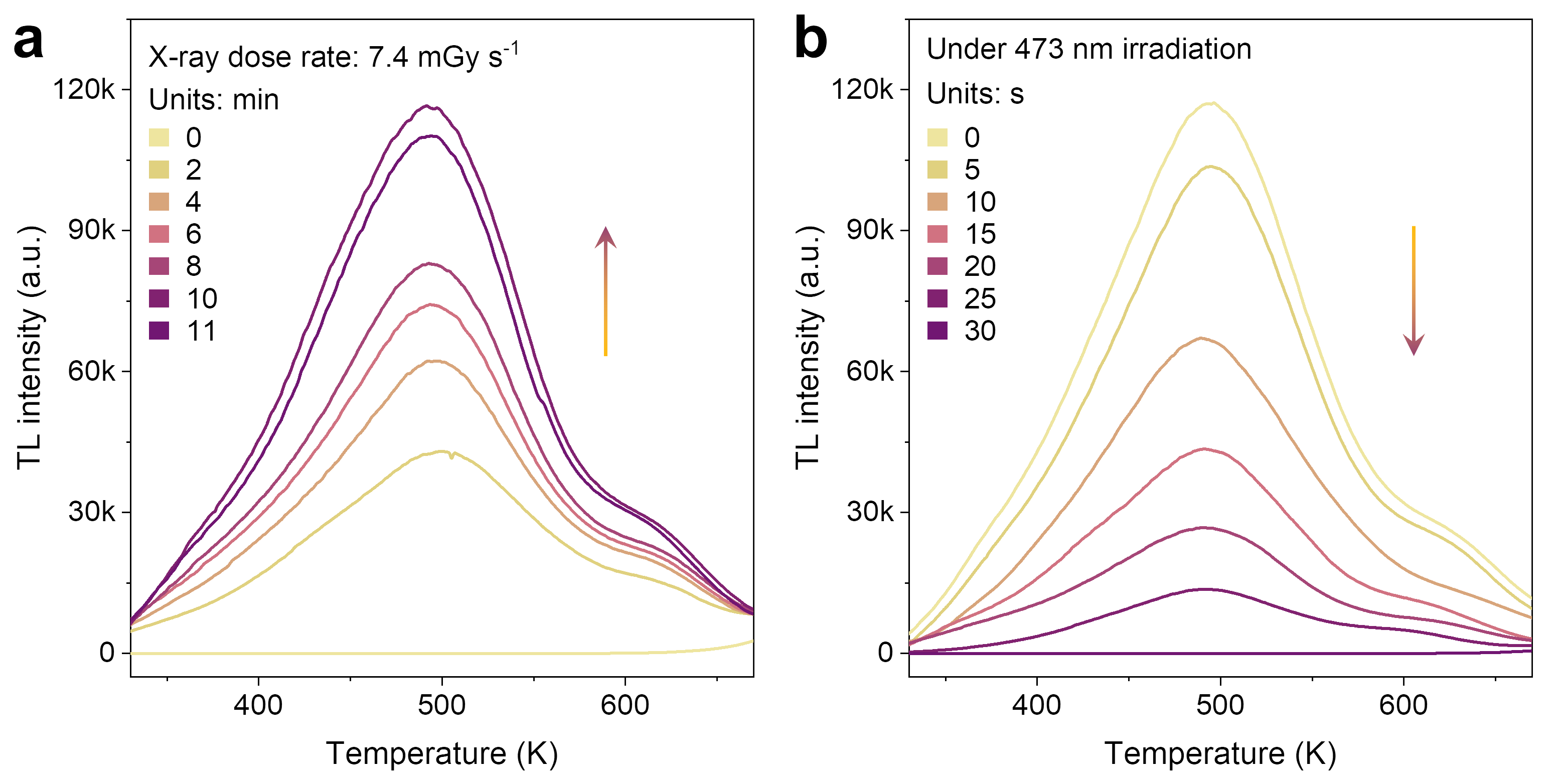


**Figure S8. a)** TL spectrum of CAS-Tb under X-ray irradiation (7.4 mGy s^-1^) for different durations; **b)** TL spectrum of CAS-Tb under 473 nm irradiation for different durations.


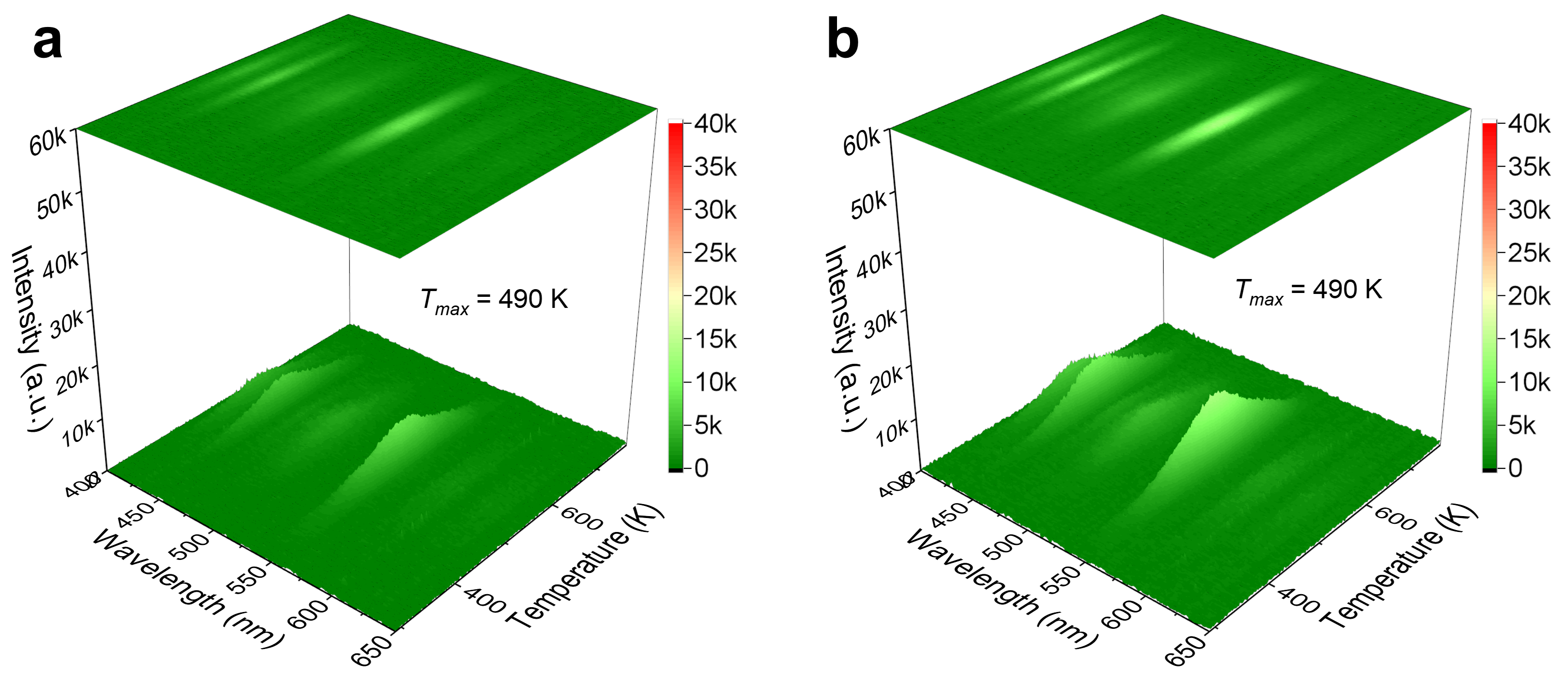


**Figure S9. a)** 3D-TL spectra and contour plots of CAS-Tb after X-ray (7.4 mGy s^-1^, 1 min) irradiation; **b)** 3D-TL spectra and contour plots of CAS-Tb after X-ray (7.4 mGy s^-1^, 2 min) irradiation.


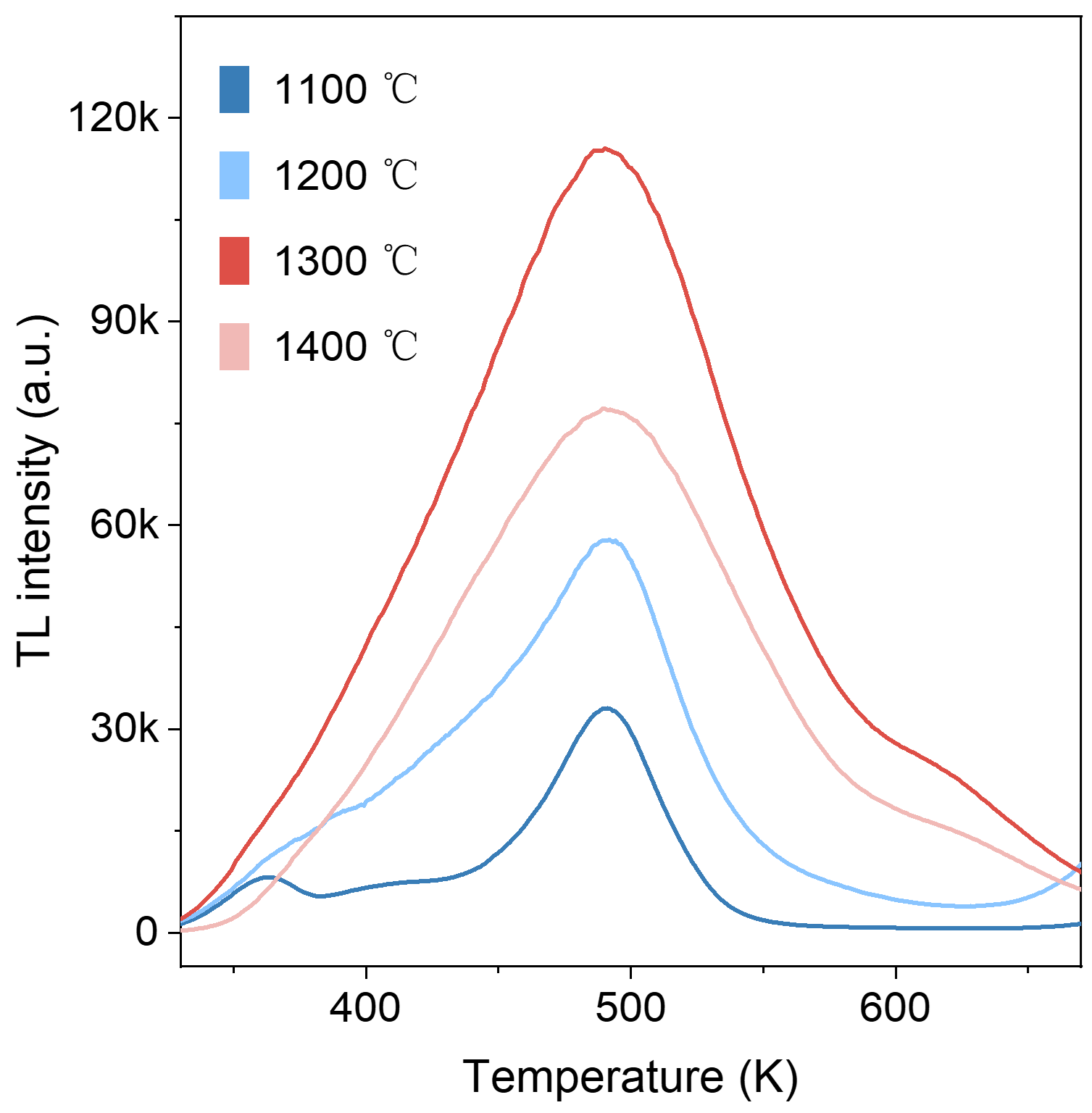


**Figure S10.** TL spectrum of CaAl_2_Si_2_O_8_: 2 mol% Tb^3+^ phosphors sintered at 1100, 1200, 1300 and 1400 ℃ under X-ray irradiation (7.4 mGy s^-1^, 10 min).


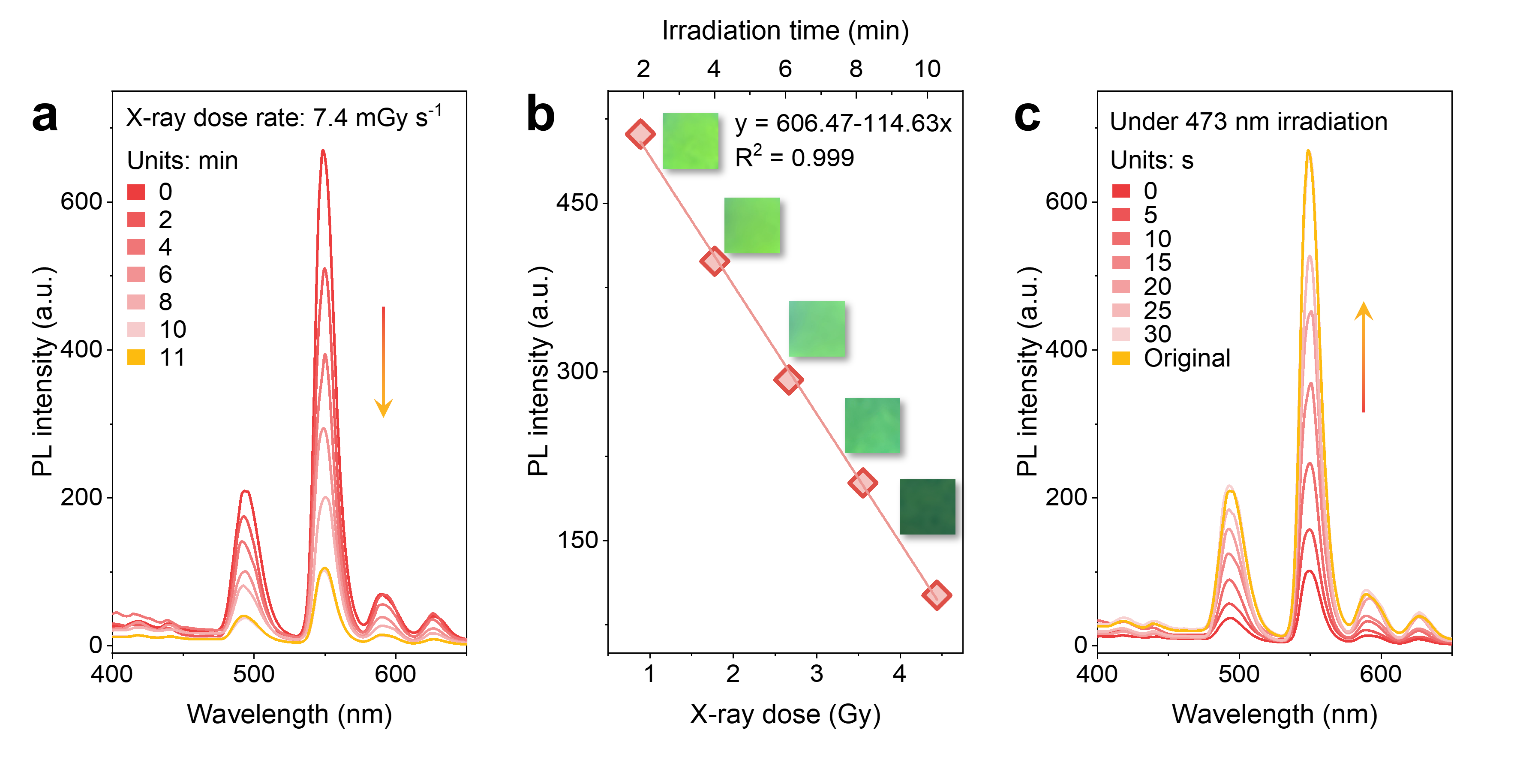


**Figure S11. a)** PL spectra of CAS-Tb under X-ray irradiation (7.4 mGy s^-1^) for different durations; **b)** Linear relationship between PL intensity and X-ray irradiation time; **c)** PL spectra of CAS-Tb under 473 nm irradiation for different durations.


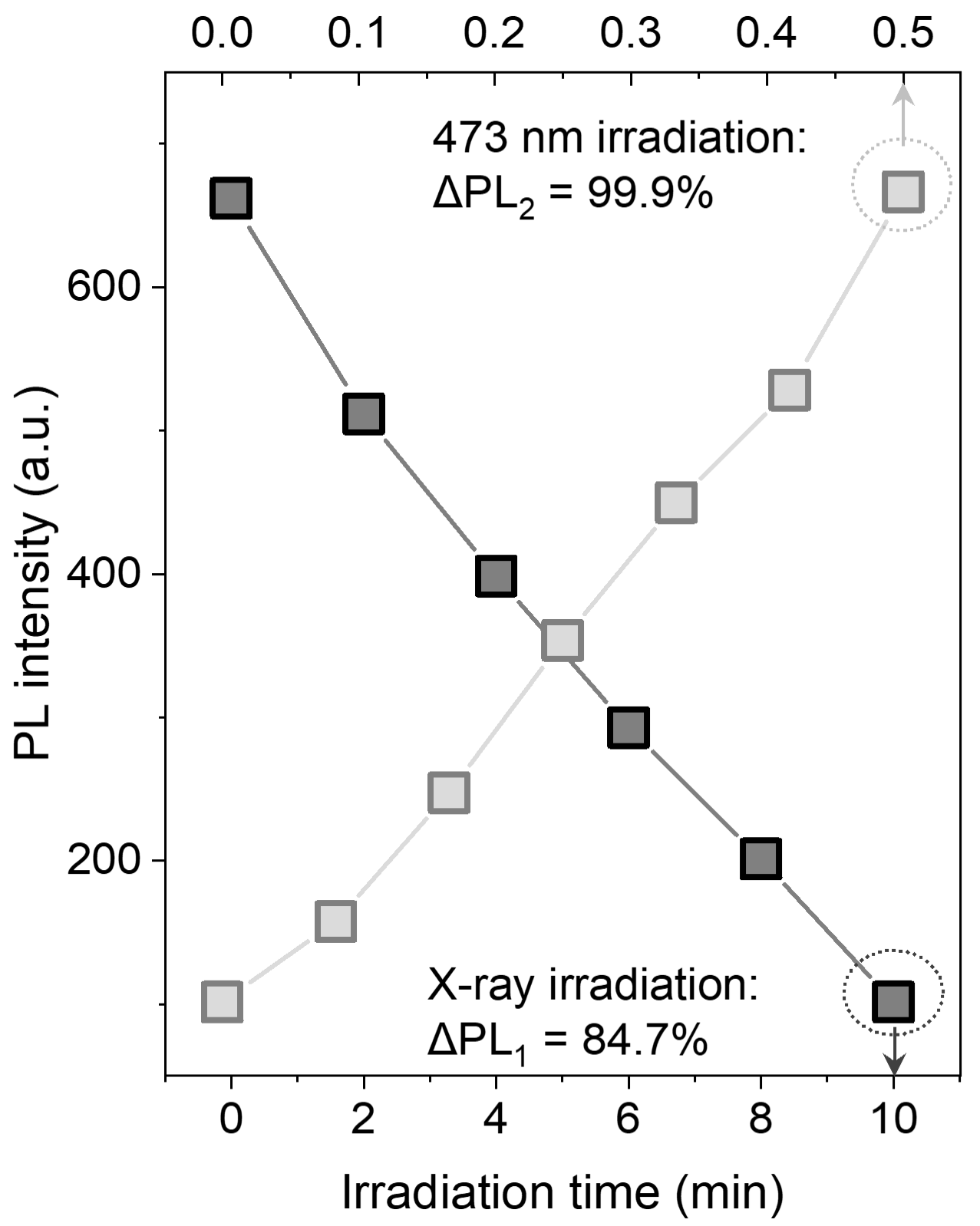


**Figure S12.** PL intensity changes at 550 nm peaks under X-ray and 473 nm laser irradiation for different times.


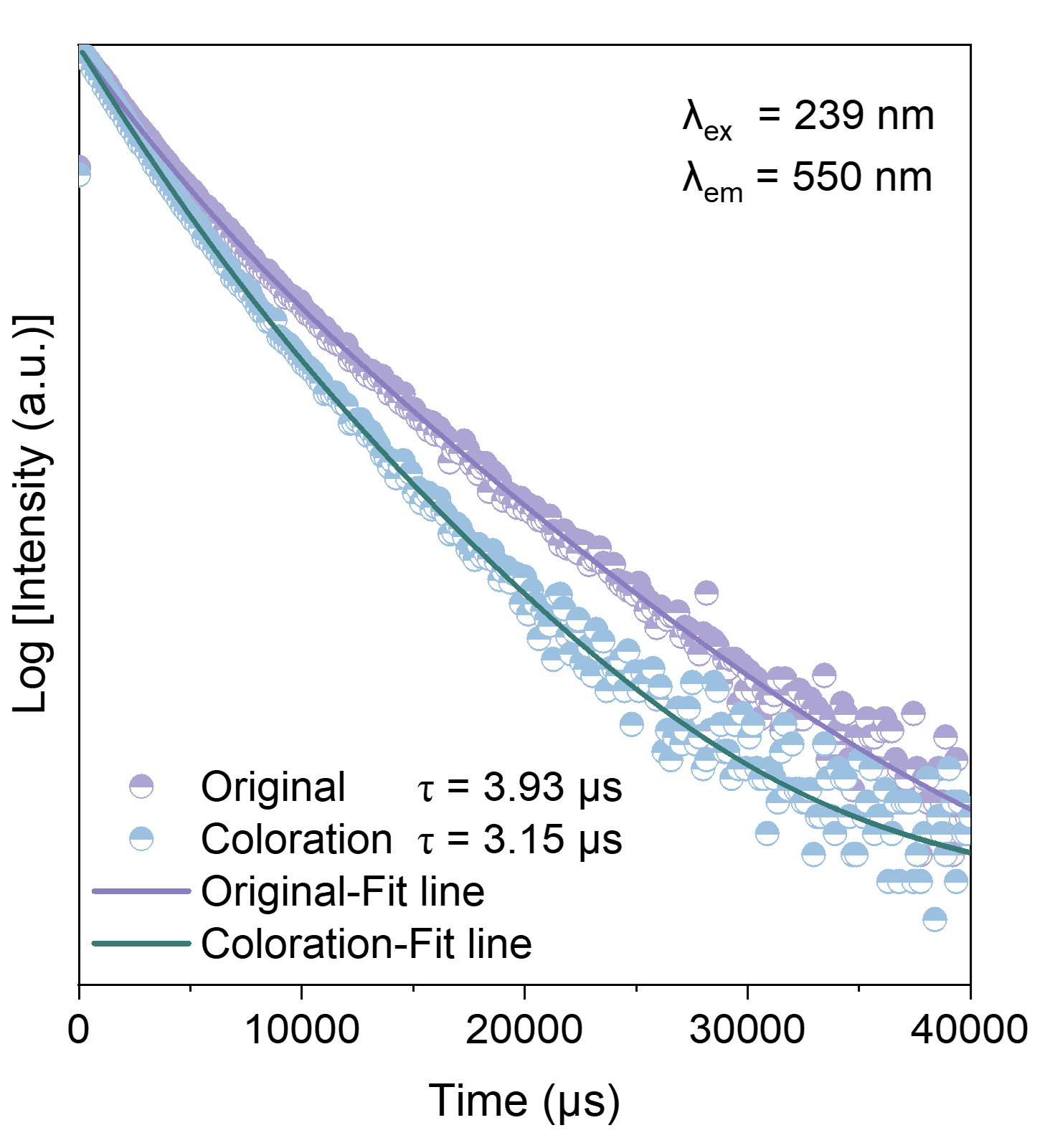


**Figure S13.** PL decay curves of CAS-Tb in original and photochromic states.


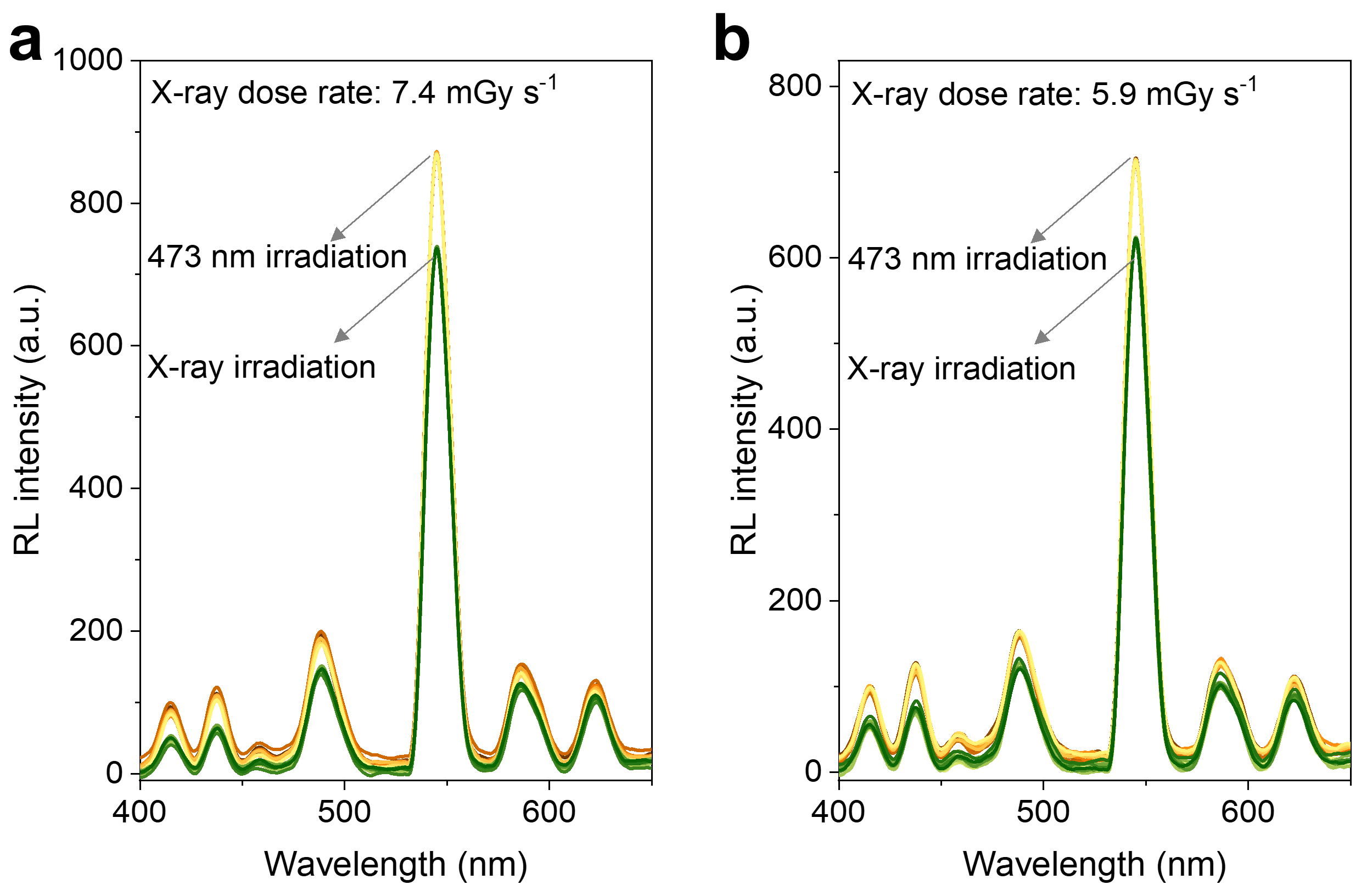


**Figure S14.** a) RL spectrum of CAS-Tb after alternating X-ray (7.4 mGy s^-1^) and 473 nm laser irradiation; b) RL spectrum of CAS-Tb after alternating X-ray (5.9 mGy s^-1^) and 473 nm laser irradiation.

References

[1] S.-B. Xiao, X. Zhang, X. Mao, H.-J. Yang, Z.-N. Chen, L.-J. Xu, *Adv. Funct. Mater.* **2024**, 34, 2404003.

[2] X. Bai, Z. Xu, Y. Zi, H. Zhao, B. Zhu, R. Feng, Y. Cun, A. Huang, Y. Liu, Y. Li, J. Qiu, Z. Song, S. J. Langford, J. Liao, Z. Yang, *Adv. Funct. Mater.* **2024**, 34, 2402452.

[3] Z. Yang, J. Hu, D. V. d. Heggen, A. Feng, H. Hu, H. Vrielinck, P. F. Smet, D. Poelman, *Adv. Funct. Mater.* **2022**, 32, 2201684.

[4] Y. Song, H. Zhao, Y. Zi, J. Qiu, Z. Song, X. Bai, J. Liao, Z. Yang, *ACS Energy Lett.* **2023**, 8, 2232.

[5] a) B. Wang, J. Peng, X. Yang, W. Cai, H. Xiao, S. Zhao, Q. Lin, Z. Zang, *Laser Photonics Rev.* **2022**, 16, 2100736; b) N. Li, Z. Xu, Y. Xiao, Y. Liu, Z. Yang, S. Liu, *Adv. Opt. Mater.* **2022**, 10, 2102232.

[6] a) X. Ou, X. Qin, B. Huang, J. Zan, Q. Wu, Z. Hong, L. Xie, H. Bian, Z. Yi, X. Chen, Y. Wu, X. Song, J. Li, Q. Chen, H. Yang, X. Liu, *Nature* **2021**, 590, 410; b) Y. Wang, D. Chen, Y. Zhuang, W. Chen, H. Long, H. Chen, R.-J. Xie, *Adv. Opt. Mater.* **2021**, 9, 2100624; c) L. Lei, Y. Wang, W. Xu, R. Ye, Y. Hua, D. Deng, L. Chen, P. N. Prasad, S. Xu, *Nat. Commun.* **2022**, 13, 5739.
